# Local Search with a SAT Oracle for Combinatorial Optimization

**DOI:** 10.1007/978-3-030-72013-1_5

**Published:** 2021-02-26

**Authors:** Aviad Cohen, Alexander Nadel, Vadim Ryvchin

**Affiliations:** 8 Eindhoven University of Technology, Eindhoven, The Netherlands; 9 Aalborg University, Aalborg East, Denmark; Intel Corporation, P.O. Box 1659, Haifa, 31015 Israel

## Abstract

NP-hard combinatorial optimization problems are pivotal in science and business. There exists a variety of approaches for solving such problems, but for problems with complex constraints and objective functions, local search algorithms scale the best. Such algorithms usually assume that finding a non-optimal solution with no other requirements is easy. However, what if it is NP-hard? In such case, a SAT solver can be used for finding the initial solution, but how can one continue solving the optimization problem? We offer a generic methodology, called *Local Search with SAT Oracle (*LSSO*)*, to solve such problems. LSSO facilitates implementation of advanced local search methods, such as variable neighbourhood search, hill climbing and iterated local search, while using a SAT solver as an oracle. We have successfully applied our approach to solve a critical industrial problem of cell placement and productized our solution at Intel.

### 1 Introduction

Real-life *combinatorial optimization problems* are pivotal in science, operations research, engineering, economics, and business [11, 13, 20, 21, 23].

Loosely speaking, an instance of a combinatorial optimization problem deals with the minimization of an objective function over a finite set, subject to *feasibility constraints* (or, simply, *constraints*). The set of all elements satisfying the constraints is referred to as the set of *feasible solutions* (or, simply, *solutions*). In this paper, we focus on solving any problem, which can be expressed as a constraint optimization program (COP) [2]. Arguably, the vast majority of combinatorial problems, encountered in practice, fall under this category.

Many important combinatorial problems are NP-hard. For such problems, various algorithmic strategies have been devised, including complete methods, such as branch-and-bound and dynamic programming, and incomplete methods, such as greedy algorithms and local search. Each such method imposes requirements on the mathematical properties of the problem with a consequent limit on the scope of applicability.

Local search algorithms stand out from the rest in that they impose relatively mild constraints on the type of the problem to be addressed, thus providing a wide scope of applicability. Furthermore, they seem to scale better with input size relative to complete algorithms [24]. This makes local search algorithms an attractive choice. However, local search algorithms may return a low-quality solution or no solution at all, given a problem for which the mere task of finding a feasible solution is NP-hard. Henceforth, we shall refer to such problems as *NP-Hard-Feasible* problems.

This paper introduces the *Local Search with SAT Oracle (*`LSSO`*)* methodology, that is, local search algorithms which use a SAT solver (or a SAT-based optimization algorithm; details appear later) as an oracle. A key advantage of our approach is that it can handle problems with complex constraints and objective functions. In particular, it can handle NP-Hard-Feasible problems.

To see how SAT solvers might be useful, consider the basic version of a local search for an optimal solution. At the beginning, the local search generates an initial solution and sets it as the current solution. Then, it enters a loop. In each iteration, it looks for a solution with a lower value of the objective function *within a neighbourhood* of the current one. If such a solution is found, it is set to be the current solution, and the execution resumes. Otherwise, the algorithm terminates and returns the current solution.

A key component of local search algorithms is the *neighbourhood function*, which assigns to each feasible solution a subset of feasible solutions, called its *neighbourhood*. Ordinarily, a neighbourhood of the current feasible solution comprises a set of solutions which can be obtained from the current solution by applying a small collection of *feasibility-preserving* perturbations to its combinatorial structure. A key concern is ensuring that neighbourhoods: (i) are polynomially searchable, and (ii) contain high-quality solutions. However, meeting *both* requirements might be challenging, since polynomial searchability implies that neighbourhoods should be small, and hence less likely to contain high-quality solutions. In addition, in the case of NP-Hard-Feasible problems, it is not clear how to achieve polynomial searchability, since a search should, in particular, be able to find a feasible solution, which is NP-hard.

Our main idea is to let the SAT solver both find an initial solution and conduct the neighbourhood search. The designer can now define feasibility constraints and neighbourhoods declaratively, that is, by a set of SAT constraints. The designer has more freedom to choose neighbourhoods, which need neither be small, nor contain only solutions close to the current solution. This is because the search of the now complex and possibly large neighbourhoods is entrusted to SAT solvers, constructed precisely to efficiently search large complex subspaces. Our approach lends itself to implementations of advanced local search variants, such as variable neighbourhood search, hill climbing and iterated local search [29].

An important feature of our algorithms is that they are *anytime*. Recall that an anytime algorithm is expected to return a valid solution even if interrupted. An anytime algorithm for an optimization problem is expected to find an *improving* set of solutions. The anytime property is essential for industrial application, since it allows the user to get an approximate solution even for very difficult instances [14, 15].

We demonstrate the usefulness of our approach by solving hard industrial instances of the NP-Hard-Feasible cell placement problem. Cell placement is one of the most important problems in VLSI automation [28]. Its most basic version concerns placing without overlap a set of rectangles on a grid, while minimizing the occupied area. In reality, the problem is more complex. Our approach has been successfully productized at Intel.

The rest of this paper is organized as follows: Sect. 2 provides the necessary background. Sect. 3 introduces our `LSSO` methodology. Sect. 4 shows how to solve placement with `LSSO`. Sect. 5 presents the experimental results. Sect. 6 concludes our paper.

### 2 Background

This section provides some background. Sect. 2.1 is an overview of COP. Sect. 2.2 describes the cell placement problem and shows how to reduce it to COP. Sect. 2.3 discusses how one can solve a COP using a SAT-based bit-vector solver. Sect. 2.4 reviews local search.

#### 2.1 Constraint Optimization Program (COP)

This work presents a new methodology for solving a wide class of combinatorial optimization problems, which can be expressed as a Constraint Optimization Program, shown in Def. 1.

**Definition 1 (Constraint Optimization Program (COP) [2]).** *A constraint optimization program is a tuple* $(\mathcal{X}, \mathcal{D}, \mathcal{C}, \Psi)$ *where:*

1. $\mathcal{X} = \{x_1 \ldots x_n\}$ *is a finite set of variables often referred to as* decision variables.
2. $\mathcal{D} = \{\mathcal{D}_1 \ldots \mathcal{D}_n\}$ *is a corresponding set of* finite *domains. Without loss of generality, each* $\mathcal{D}_i$ *is assumed to be a closed bounded interval of non-negative integers.*
3. $\mathcal{C} = \{\mathcal{C}_1 \ldots \mathcal{C}_m\}$ *is a finite set of constraints* $\mathcal{C}_k : \mathcal{D}_1 \times \cdots \times \mathcal{D}_n \mapsto \{0, 1\}$.
4. $\Psi : \mathcal{D}_1 \times \cdots \times \mathcal{D}_n \mapsto \mathbb{Z}$ *is an objective function to be minimized.*

#### 2.2 The Cell Placement Problem

*Cell Placement (Placement)* is a major stage in the VLSI design cycle [8,16]. The input of the cell placement problem comprises the following components:

1. A *rectangular grid region* of $\boldsymbol{M}$ rows and $\boldsymbol{N}$ columns, on which the cells are to be placed. Row/column line numbering starts at 0 and ends at $\boldsymbol{M}/\boldsymbol{N}$, respectively.
2. A finite set $\mathcal{C}$ of *rectangular cells*. The width and the height of each cell $c \in \mathcal{C}$ are assumed to be positive integers, denoted by $c^{width} : 0 < c^{width} \leq \boldsymbol{N}$ and $c^{height} : 0 < c^{height} \leq \boldsymbol{M}$, respectively.
3. A set $\mathcal{R}$ of *forbidden rectangular regions*. A forbidden region $r \in \mathcal{R}$ is specified by 4 numbers $r^{west}$, $r^{south}$, $r^{east}$ and $r^{north}$ (where, $0 \leq r^{west}, r^{east} < \boldsymbol{N}; 0 \leq r^{south}, r^{north} < \boldsymbol{M}; r^{east} > r^{west}; r^{north} > r^{south}$), denoting the leftmost column line, bottom row line, rightmost column line, and top row line, respectively.
4. A finite set $\mathcal{I}$ of *nets*, each consisting of a non-empty subset of cells. The nets may (and usually do) intersect.

We are interested in *feasible* placements, that is, placements in which no cell overlaps other cells or forbidden regions. Given a feasible placement, we define the *size of a net* $n \in \mathcal{I}$ as the perimeter of the box bounding its placed cells. We define the *size of the placement* as the sum of the sizes of the nets. We are required to find a feasible placement of a minimal size. An example is shown in Fig. 1.

In industrial practice, there may be additional *industrial requirements*, such as aligning some of the cells, enforcing parity constraints (i.e., the user might require the $y$ coordinates of some of the cells to be either even or odd) [19], ensuring a minimal distance between some of the cells and others. We omit further details due to IP considerations.

Placement is NP-Hard-Feasible, since the NP-complete bin packing problem can be reduced to the decision version of the placement problem [10].

**2.2.1 Constraint Optimization Program for Cell Placement.** We show how to construct a COP for the cell placement problem. For each cell $c \in \mathcal{C}$, let $c^{west}$ and $c^{east}$ denote its leftmost and rightmost column respectively, and $c^{south}$ and $c^{north}$ denote its bottom and top row. Strictly speaking, it suffices to use $c^{west}$ and $c^{south}$ as the COP's independent variables, but it is convenient to use $c^{east}$ and $c^{north}$ as syntactic sugar for $c^{west} + c^{width}$ and $c^{south} + c^{height}$, respectively. The COP looks as follows:

1. *Variables*: $\{c^{west}, c^{south} \mid c \in \mathcal{C}\}$
2. *Domains*: $c^{west} \in [0 \ldots \boldsymbol{N} - 1]$ and $c^{south} \in [0 \ldots \boldsymbol{M} - 1]$
3. *Feasibility constraints*:
   (a) Each cell $c$ is placed wholly within the grid region:
   $$(c^{west} \geq 0) \wedge (c^{east} \leq \boldsymbol{N}) \wedge (c^{south} \geq 0) \wedge (c^{north} \leq \boldsymbol{M})$$
   (b) For every pair of cells $\langle c_i, c_j \rangle$, such that $i < j$, there is no overlap:
   $$(c_i^{west} \geq c_j^{east}) \vee (c_j^{west} \geq c_i^{east}) \vee (c_i^{south} \geq c_j^{north}) \vee (c_j^{south} \geq c_i^{north})$$
   (c) For every pair $\langle r, c \rangle$ of a forbidden region $r$ and a cell $c$, there is no overlap:
   $$(r^{west} \geq c^{east}) \vee (c^{west} \geq r^{east}) \vee (r^{south} \geq c^{north}) \vee (c^{south} \geq r^{north})$$
   (d) Constraints representing any additional industrial requirements.
4. *Objective function $\Psi$*: for every net $n \in \mathbb{I}$, let $\|n\|$ denote its size. We have:

$$\|n\| = \left(\max_{c \in n}(c^{east}) - \min_{c \in n}(c^{west})\right) + \left(\max_{c \in n}(c^{north}) - \min_{c \in n}(c^{south})\right)$$
$$\Psi = \sum_{n \in \mathbb{I}} \|n\|$$

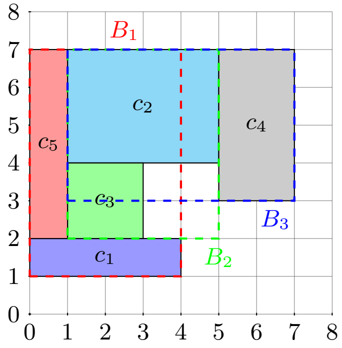

Fig. 1: Placement example [16]. A solution is shown for the problem of placing five cells $c_1$, $c_2$, $c_3$, $c_4$ and $c_5$ of sizes $4 \times 1$, $4 \times 3$, $2 \times 2$, $2 \times 4$ and $1 \times 5$ respectively, on a grid with $\boldsymbol{M} = \boldsymbol{N} = 8$. There are three nets: $n_1 = \{c_1, c_3, c_5\}$, $n_2 = \{c_2, c_3\}$ and $n_3 = \{c_2, c_4\}$ (without any forbidden regions). The bounding boxes of the nets are $B_1$, $B_2$ and $B_3$, respectively. The sizes of the nets, comprising the perimeters of the bounding boxes, are 20, 18 and 20, respectively. The overall placement size is $20 + 18 + 20 = 58$. The solution is an optimal one.

#### 2.3 Solving COP with SAT

A COP can be solved with various types of solvers [2]. In particular, it is possible to solve a COP by reduction to a series of SAT solver invocations through bit-vector reasoning as explained below.

**2.3.1 Bit-vector Solving and SAT.** We start with reviewing the basic terminology, related to SAT solving. A *literal* $l$ is a Boolean variable $v$ or its negation $\neg v$. A clause is a disjunction of literals. A formula $F$ is in *Conjunctive Normal Form (CNF)* if it is a conjunction (set) of clauses.

A SAT solver [4] receives a CNF formula $F$ and returns a satisfying assignment (aka, model or solution), if one exists. In *incremental SAT solving under assumptions* [5, 18], the user may invoke the SAT solver multiple times, each time with a different set of *assumption literals* (called, simply, the *assumptions*) and, possibly, additional clauses. The solver then checks the satisfiability of all the clauses provided so far, while enforcing the values of the current assumptions.

A *bit-vector variable (bit-vector)* of *width* $n = |B|$, $B = \{v_n, v_{n-1}, \ldots, v_1\}$, is a sequence of $n$ Boolean variables, called *bits*. Bit $v_1$ is the Least Significant Bit (LSB) and $v_n$ is the Most Significant Bit (MSB). A *Boolean constant* is either $\bot$ (0) or $\top$ (1). A *bit-vector constant* is a bit-vector (BV), each one of whose bits is substituted by a Boolean constant. A *bit-vector term* is either a bit-vector, a BV constant, or a result of applying an operator which returns a bit-vector (for example, BV addition, if-then-else, concatenation) over other terms and atoms. An *atom* is either a Boolean variable, a Boolean constant or a result of applying an operator, which returns a Boolean (for example, $=$ or unsigned-less-than), over BV terms and atoms. A *bit-vector formula* (also known as a *bit-vector constraint*) is recursively defined to be either an atom, a negation of a bit-vector formula, or the result of applying the Boolean operator $\wedge$ or the Boolean operator $\vee$ over two or more bit-vector formulas. See [3, 12] for a rigorous description of the BV language. A BV solver decides the satisfiability of BV formulas.

A BV formula $F$ is *satisfiable* iff it has a *model*, that is, an assignment of BV and Boolean constants to their corresponding BV and Boolean variables, which satisfies $F$. In this paper, BV constants are interpreted as unsigned numbers, and BV comparison operators are interpreted as unsigned. For example, given a bit-vector $B = \{v_3, v_2, v_1\}$, the formula $F = B < 2$ has two models $\mu_1 : \mu_1(B) = 0$ and $\mu_2 : \mu_2(B) = 1$.

All the algorithms presented in this work are assumed to use the so-called *eager* BV solver [6] which, following some preprocessing, translates the input BV formula to an equisatisfiable formula in CNF and solves it with a SAT solver. Thus, we will use the notions of BV solving and SAT solving interchangeably. We also assume the BV solver to have the same incremental API as a SAT solver.

Since the variables in a COP have finite domains, both the variables and the constraints of a COP can be easily expressed as BV variables and BV constraints.

In particular, in the COP constructed for the cell placement problem in Sect. 2.2.1, the variables and the constraints can be expressed as BV variables and constraints as follows: For each cell $c$, we define four bit-vectors: $c^{west}$ and $c^{east}$ of width $\lceil \log N \rceil$ as well as $c^{south}$ and $c^{north}$ of width $\lceil \log M \rceil$. All the constraints in our COP involve these bit-vectors and can be expressed in terms of operators and relations available in the BV language [3]. Specifically, we implement min and max operators using a series of if-then-else operators. In addition, for every operator, we zero-extend the widths of the operands and the resulting bit-vector to prevent an overflow, whenever required.

Reducing the constraints of a COP to a BV formula and invoking BV solver suffices to find one non-optimal solution. However, for solving the optimization problem by reduction to BV, one needs an extension of BV solving to optimization.[1]

**2.3.2 Extending Bit-vector Solving to Optimization.** One can extend bit-vector solving to the so-called Bit-Vector Optimization (OBV) [19] as follows:

A model $\mu$ of a BV formula $F$ is *$T$-minimal*, for a given bit-vector $T$, iff $\mu(T) \leq \nu(T)$ (where the comparison is unsigned) for every model $\nu$ of $F$. Given a BV formula $F$ and a term $T = \{t_n, t_{n-1}, \ldots, t_1\}$ in $F$, where $T$ is called the *optimization target* (or, simply, the *target*), *Bit-Vector Optimization (OBV)* is the problem of finding a $T$-minimal model of $F$. The bits of the target $T$ are referred to as the *target bits*.

Translating our placement COP to OBV is straightforward. We have already shown how to translate the constraints. The optimization target is constructed in the same way as the objective function $\Psi$ is constructed in the COP.

How can one solve OBV in practice? First, one can use the following simple anytime Linear Search algorithm, implemented on top of an incremental BV solver [16,27]:

$solver.Assert(F)$; $\mu := solver.Sat()$ ▷ assert $F$ and find the first solution
**while** $\mu$ is a solution **do** ▷ while there is still a solution
  $solver.Assert(T < \mu(T))$ ▷ block all the solutions with cost $\geq \mu(T)$
  $\mu := solver.Sat()$ ▷ can we improve our solution?
**return** $\mu$ ▷ $\mu$ is guaranteed to be $T$-minimal

Another anytime algorithm to solve OBV is the following binary search-based algorithm, called `OBV-BS` [9,19]:

$solver.Assert(F)$; $\mu := solver.Sat()$ ▷ assert $F$ and find the first solution
$i := n$ ▷ $i$ is the current bit number, initialized to the MSB
**while** $i \geq 1$ and $\mu(t_i) = \bot$ **do** ▷ fix to $\bot$ the MSBs, assigned to $\bot$ in $\mu$
  $solver.Assert(\neg t_i)$
  $i := i - 1$ ▷ after the loop, $i$ will point to the first target bit, assigned $\top$
**while** $i \geq 1$ **do** ▷ Check one-by-one, if we can flip the remaining target bits to $\bot$
  $\mu := solver.Sat(\{\neg t_i\})$ ▷ run the solver under the assumption $\neg t_i$
  **if** satisfiable **then**
    **while** ($i \geq 1$ and $\mu(t_i) = \bot$) **do** $solver.Assert(\neg t_i); i := i - 1$ **endwhile**
  **else**
    $solver.Assert(t_i); i := i - 1$ ▷ $t_i$ cannot be flipped to $\bot$, so we fix it to $\top$
**return** $\mu$

We have successfully applied `OBV-BS` for solving the problem of *fixing* an existing placement [19], closely related to the generic placement problem, we are exploring

[1] One cannot use MaxSAT [26]–the widely used extension of SAT to optimizing a linear Pseudo-Boolean (PB) function–to solve COP in the generic case, since the objective function is not guaranteed to be linear PB. In particular, it is *not* linear PB for placement, if only because the variables are bit-vectors, rather than Booleans.

in this work. However, both Linear Search and OBV-BS failed to scale to industrial instances of our current problem of finding an optimal placement from scratch (with Linear Search scaling somewhat better than OBV-BS).

Recently, we have introduced the so-called Polosat anytime algorithm [16], which can be used *instead* of the standard SAT solver inside Linear Search (and other SAT-based anytime optimization algorithms) to make it substantially more scalable. The idea behind Polosat, shown below, is to simulate local search using a SAT solver. We use the strictly-monotone version of Polosat [16], which assumes the availability of the so-called Boolean *observable variables (observables) Obs*, that is, a set of Boolean variables on which the objective function depends (for placement, the observables might comprise the bits of the bit-vectors, representing the sizes of the nets, for every net). Polosat is carried out by getting a model $\mu$ and then trying to improve it by repeatedly flipping observables, which have not been assigned $\bot$ in previous models:

**function** SOLVER.POLOSAT(assumptions)
**Require:** Target bit-vector $T$ is available; Observables *Obs* are available.
    $\mu := solver.Sat(assumptions)$ ▷ get the first model $\mu$
    *is_good_epoch* $:= 1$ ▷ good epoch: an iteration, which improves $\mu$
    **while** *is_good_epoch* **do** ▷ one loop is an epoch
        $B := \{v : v \in Obs, \mu(v) = \top\}$ ▷ remove any observables, assigned $\bot$
        *is_good_epoch* $:= 0$
        **while** $B$ is not empty **do**
            $b_i :=$ *B.front*(); *B.dequeue*()
            $\sigma := solver.Sat(assumptions \cup \{\neg b_i\})$ ▷ trying to flip $b_i$
            **if** satisfiable **then**
                **if** $\sigma(T) < \mu(T)$ **then** $\mu := \sigma$ and *is_good_epoch* $:= 1$
            $B := \{b : b \in B, \sigma(t) = 1\}$ ▷ remove any observables, assigned $\bot$
    **return** $\mu$

To combine Polosat into Linear Search, it is sufficient to replace solver.Sat invocations by solver.Polosat invocations in the code. [2] We have shown in [16] that replacing plain SAT invocations by Polosat invocations in Linear Search makes our placement tool substantially more scalable. We reaffirm this result in Sect. 5.

Yet, despite the significant progress we had witnessed when applying Polosat, we found that combining Polosat into Linear Search is still insufficient for solving a variety of complex real-world instances of our industrial placement problem. This empirical challenge lead us to develop our LSSO methodology, presented in this paper. As we shall see, combining LSSO and Polosat makes our tool considerably more scalable, while the methodology itself is generic and can be applied to solving a wide range of optimization problems.

### 2.4 Local Search Algorithms

Local search strategies [1] are a collection of *algorithmic templates*. An algorithmic template specifies the main flow of an algorithm, but leaves some details unimple-

[2] Polosat also uses polarity fixing strategies, such as TORC [14,17], omitted here; please refer to [16] for details. Additional non-anytime OBV algorithms are introduced in [19,22].

mented. By implementing these details for a specific problem, one obtains an algorithmic solution for that problem.

**2.4.1 Basic Local Search Strategy.** The basic strategy generates an initial feasible solution and sets it as the *current solution*. Then, it enters a loop. In each iteration, it looks within a *neighbourhood* of the current solution for a feasible solution with a lower value of the objective function. If one is found, it is set to be the current solution. Otherwise, the algorithm is terminated returning the current solution. Note that this version is guaranteed to stop; it does so, when it reaches a *local minimum* of the objective function with respect to the neighbourhood used.

To turn this algorithmic template into a complete algorithm, one has to implement the following *problem-dependent* items: (i) A procedure for generating an initial feasible element. (ii) A *neighbourhood function* assigning to each solution a subset of solutions. (iii) An algorithm for searching the neighbourhood for a better solution.

**2.4.2 Neighbourhood Functions.** A key factor, which affects both the complexity of the search and the quality of the resulting solution, is the selection of a *neighbourhood function*. In theory, the selection ought to depend on a mathematical analysis of the structure of the feasible set and the profile of the objective function. For complex problems, such an analysis is usually beyond reach. The classical approach to neighbourhood definition is based on the following problem-independent general principles:

1. Drawing on an analogy to optimization algorithms in the continuous case (such as gradient descent or line search), a neighbourhood should be so defined as to make its elements "close" to the current solution. So, typically, the neighbourhood of a feasible solution is specified by a small class of *feasibility-preserving* modifications/perturbations to its combinatorial structure.
2. A neighbourhood should be so defined as to ensure that it is *polynomially searchable*. Hence, unless we have a sophisticated non-exhaustive neighbourhood search algorithm, neighbourhoods should be small.

However, as we have argued in Sect. 1, this approach is not without issues. In particular, feasibility-preserving perturbations may not be easy to find, especially for NP-Hard-Feasible problems, while having small neighbourhoods implies a low likelihood of high-quality solutions.

**2.4.3 Advanced Versions of Local Search.** A disadvantage of the basic version of local search is that it may stop at a local minimum of a poor quality, if too small a region of the feasible space is explored. To circumvent this outcome, advanced variants enabling an exploration of larger portions of the feasible space have been devised [7, 29]. Those described here provide some mechanism to escape from the local minimum to "nearby" solutions and resume the search from there. They have been designed to accommodate situations, where local minima are not distributed uniformly in the feasibility space, but are rather clustered in close proximity [25].

The *variable neighbourhood search* approach uses multiple neighbourhoods to escape from local minima. It relies on the fact that a local minimum with respect to one neighbourhood need not be a local minimum with respect to another (if the latter is not contained in the former). The algorithm maintains a set of neighbourhood functions. Once a local minimum with respect to the current neighbourhood is reached, the neighbourhood is switched, and the search is resumed.

The *hill climbing* method allows the selection of a non-improving solution, once a local minimum is reached. Since the objective function no longer monotonically decreases, there is now a possibility of a cycle: a solution may be visited more than once forcing the search into an infinite loop. One can deal with this problem in various ways: ignore it and let the algorithm run until the timeout expires, use randomization, or introduce data structures that keep track of the search history and prohibit solutions that have already been encountered. The latter approach is referred to as *tabu search*.

Another idea is to use *large neighbourhoods*. This approach increases the size of the explored region and the likelihood of better solutions. However, large neighbourhood search may become intractable.

The *iterated local search* approach can be viewed as "a local search within a local search". In each iteration of the search, it uses a *subsidiary search algorithm* to explore iteratively a feasible sub-space. Once a local minimum is returned, a new search is initiated in a region, whose elements are obtained by "perturbing" the recent solution.

All the above approaches can be implemented within our `LSSO` framework. The key difference between `LSSO` and previous approaches is using SAT or `Polosat` as an oracle for both finding the initial solution and carrying out the neighbourhood search.

### 3 Local Search with SAT Oracle (`LSSO`)

This section introduces the main contribution of our paper. We propose using SAT as an oracle in local search algorithms to address the scalability and quality issues that arise in the classical local search algorithms, especially, given an NP-Hard-Feasible problem.

Given a combinatorial optimization problem, the first stage in designing an `LSSO` solution is expressing the problem as a COP.

In the second stage, the COP decision variables are translated to bit-vectors, and the feasibility constraints are translated to a BV formula (including any additional industrial requirements). One might experiment with several alternative formulations and select the one deemed best.

The third step is defining the so-called neighbourhood generators. A *neighbourhood generator* $\mathcal{N}(\mu)$ accepts as an input a solution $\mu$ (that is, a model to the bit-vector formula, representing the COP), and generates *neighbourhood constraints*. The set of all the assignments which satisfy the feasibility and neighbourhood constraints constitutes the neighbourhood of the solution. Thus, finding such an assignment amounts to finding an element of the neighbourhood of $\mu$.

A key ingredient of our methodology is the adoption of a neighbourhood concept, which differs significantly from the classical one, described in Sect. 2.4.2:

1. The neighbourhood need not be small and need not contain (only) elements "close" to the current solution.

2. Normally, $\mathcal{N}(\mu)$ should generate constraints which ensure a cost lower than that of $\mu$. If such a formulation is possible, then an iteration of the local search algorithm merely needs to find a model to these constraints in order to progress.
3. If the objective function is too complex to model in its entirety, a neighbourbood generator might attempt to ensure a better value for the objective function by imposing constraints on the objective function's sub-components. For example, when the objective function is a very large sum of bit-vector terms, one might impose constraints on the sum's terms or small partial sums thereof.
4. Notwithstanding the above, neighbourhood generators may support *hill climbing*, in which case, the constraints are so formulated as to admit non-improving solutions.

Note that, in our approach, neighbourhoods direct the search to "higher-quality" regions with respect to the current solution, regardless of the algorithmic difficulties of searching such regions. This is another key aspect of our approach: we trust SAT solvers to search complex sub-spaces efficiently.

Having discussed neighbourhoods, we are now ready to describe the simplest `LSSO` implementation:

1. A BV solver instance is created and the COP is provided to the solver. Specifically, we represent the COP's decision variables as bit-vectors, where the widths are chosen to accommodate the largest values. We provide the feasibility constraints to the solver as BV constraints. Then, we implement neighbourhood generators, which, given a feasible solution, return a set of BV constraints defining its neighbourhood.
2. The local search is carried out as follows:
   (a) The algorithm obtains an initial solution by asserting the feasibility constraints and asking the solver for a model. This model is set as the *current solution* $\mu$.
   (b) The algorithm enters a loop, in which the solver operates in incremental mode. In each iteration, the algorithm calls the neighbourhood generator with the current solution as input, to generate a list of BV constraints. These are provided to the solver, which is asked for a model. If a model $\alpha$ is found, $\mu$ is set to $\alpha$. Otherwise, the algorithm terminates returning $\mu$.

The neighbourhood constraints can be given to the solver as either *assumptions* or *assertions*. This leads to two types of search, providing a tradeoff between execution time and quality:

1. *Non-speculative search*: the neighbourhood constraints are passed to the solver as *assertions*. Once assertions are passed to the solver, they are enforced in all ensuing iterations. The search proceeds through a monotone sequence of decreasing neighbourhoods until a local minimum is reached. Thus, the search is localized and is relatively fast at the possible expense of quality.
2. *Speculative search*: the neighbourhood constraints are passed to the solver as *assumptions*. The neighbourhood constraints are valid only for one iteration. Thus, the current neighbourhood is not intersected with previous neighbourhoods and a larger portion of the feasibility space will be explored. The search is expected to be slower, since the SAT solver handles assumptions less efficiently than assertions [18], but the quality of resulting solution is expected to be better, since the search can explore a greater part of the feasibility space, especially so by variable neighbourhood search and hill climbing.

Alg. 1 depicts our implementation of LSSO. The algorithm receives four inputs. The Boolean inputs $\mathcal{VNS}$, $\mathcal{HC}$, and $\mathcal{SPEC}$ specify whether variable neighbourhood search, hill climbing, and speculative search are to be used. All combinations are possible, except that *hill climbing requires speculative search.* The input $\mathcal{N}_{max}$ applies to variable neighbourhood search. It specifies an upper bound on the number of consecutive neighbourhood switches without finding a solution. If that bound is exceeded, the algorithm terminates with the current solution. To effect variable neighbourhood search, the algorithm uses a predefined list of neighbourhood generators $\mathcal{N} = [\mathcal{N}_0(\mu), \mathcal{N}_1(\mu) \dots]$. The first generator $\mathcal{N}_0(\mu)$ is considered the default and is used most of the time. The others are used to escape local minima.

Alg. 1 carries out iterated local search with Polosat as an oracle, where the observables are recommended to be set to the bits of the inputs of the objective function. One can also replace the Polosat invocation by an ordinary SAT invocation.

## 4 LSSO Algorithms for the Cell Placement Problem

This section presents our LSSO-based placement algorithms. All the algorithms are instantiations of Alg. 1 with different sets of parameters. The BV constraints are generated by translating the COP constraints, as discussed in Sect. 2.3. Each algorithm uses some of the neighbourhood generators defined in Sect. 4.1.

The algorithms are presented in Sect. 4.2. None of the algorithms define the target bit-vector explicitly, since they rely on local search instead of OBV solving. By default, the algorithms use Polosat as the oracle, where the observables comprise all the bits of the bit-vectors, representing the sizes of the nets, where the size of net $n$ is given by the following bit-vector term (for every intermediate term and the resulting term $\|n\|$, its width is set to the minimal possible width which prevents an overflow, where the operators are zero-extended, whenever required):

$$\|n\| = \left(\max_{c \in n}(c^{east}) - \min_{c \in n}(c^{west})\right) + \left(\max_{c \in n}(c^{north}) - \min_{c \in n}(c^{south})\right)$$

### 4.1 Neighbourhood Generators

**4.1.1 Neighbourhood Generator $N_1$.** Let $\mu$ be a placement, that is, a model to the bit-vector formula representing the feasibility constraints. The neighbourhood $N_1(\mu)$ is designed for a highly localized fast search at the possible expense of quality. To this end, the constraints corresponding to $N_1(\mu)$ force a decrease of the objective function in a very constrained manner, so as to help the solver to come back quickly. $N_1(\mu)$ consists of all of legal placements, for which all the nets are no bigger and at least one net is smaller than under $\mu$, thus ensuring a lower cost. The constraints are:

$$\overbrace{(\bigwedge_{n \in \mathbb{I}} (\|n\| \leq \mu(\|n\|)))}^{each\ net\ is\ no\ bigger} \bigwedge \overbrace{(\bigvee_{n \in \mathbb{I}} (\|n\| < \mu(\|n\|))}^{at\ least\ one\ net\ is\ smaller}$$

**Algorithm 1** Local Search with SAT Oracle (LSSO)

```
procedure LocalSearch(VNS = ⊤, HC = ⊤, SPEC = ⊤, N_max = 10)
Require: L                                          ▷ feasibility constraints
Require: N := [N_0(μ), N_1(μ) ...]                  ▷ neighbourhood constraints generators
Require: J(x)                                       ▷ hill climbing constraints generator
        ▷ From now on, confine the search to the feasible space
    solver.Assert(L)
    current ← solver.Sat()                          ▷ find the initial solution
    if ¬current then return None                    ▷ the problem is unsatisfiable
        ▷ Loop initialization
    best ← current
    stop ← ⊥                                        ▷ stopping condition
    jump ← ⊥                  ▷ indicates whether hill climbing should be attempted
    i ← 0                                           ▷ current neighbourhood index
    while ¬stop do
        ▷ Compute neighbourhood constraints
        if HC ∧ jump then                           ▷ hill climbing is required
            neighbourhood_constraints := J(current)
        else                                        ▷ hill climbing is not required
            neighbourhood_constraints := N[i](current)
        ▷ If the mode is speculative, constraints are assumptions; otherwise they are assertions
        if SPEC then
            assertions := []; assumptions := neighbourhood_constraints
        else
            assertions := neighbourhood_constraints; assumptions := []
        ▷ Search for the next solution
        solver.Assert(assertions)
        next ← solver.Polosat(assumptions)
        if next then                                ▷ found a solution
            current ← next; i ← 0; jump ← ⊥
            if current.cost < best.cost then best ← current
            continue
        ▷ ▷ ▷ Solution not found
        ▷ If we are in variable neighbourhood mode and the number of consecutive neighbour-
        hood switches without a model has not exceeded the bound, move to next neighbourhood
        if VNS ∧ (i < (N_max − 1)) then
            i ← i + 1
            continue
        ▷ If we are in hill climbing mode, and have exhausted the bound on neighbourhood
        switches without getting a model, and hill climbing has not already been attempted in this
        iteration, attempt it in the next iteration
        if HC ∧ ¬jump then
            jump ← ⊤
            continue
        ▷ If we got here, we are stuck and need to terminate
        stop ← ⊤
    return best
```

**4.1.2 $N_2$: a Family of Neighbourhood Generators.** The $N_2$ family is designed for *variable neighbourhood search*. Each of its neighbourhoods strictly contains $N_1$ and allows the objective function to decrease in more ways. This implies higher quality solutions at the expense of slower convergence. To define the $N_2$ family, let $\alpha = \|\mathbb{I}\|$ be the number of the nets and assume $\alpha \geq 3$. For each permutation $\sigma$ of $[1 \ldots \alpha]$ and positive number $2 \leq d < \alpha$ we define a neighbourhood function $N_2[\sigma, d](\mu)$ as follows: Let $n_{\sigma(1)}, \ldots n_{\sigma(\alpha)}$ be the permuted sequence of the nets. Partition this sequence into $\lceil \alpha/d \rceil$ segments of size $d$ (last segment could be shorter). The neighbourhood $N_2[\sigma, d](\mu)$ consists of all of legal placements, for which the sum of the net sizes of each segment is no bigger than under $\mu$, and the sum of at least one segment is smaller. Note that this ensures a cost lower than the placement under $\mu$. By choosing different pairs $\langle \sigma, d \rangle$, one may obtain different neighbourhoods. The constraints are:

$$\left( \overbrace{\bigwedge_{k=1}^{\lceil \alpha/d \rceil} \left( \sum_{i=(k-1)d+1}^{\min(kd,\alpha)} \|n_{\sigma(i)}\| \leq \sum_{i=(k-1)d+1}^{\min(kd,\alpha)} \mu(\|n_{\sigma(i)}\|) \right)}^{\textit{each sum is no bigger}} \right)$$

$$\bigwedge$$

$$\left( \overbrace{\bigvee_{k=1}^{\lceil \alpha/d \rceil} \left( \sum_{i=(k-1)d+1}^{\min(kd,\alpha)} \|n_{\sigma(i)}\| < \sum_{i=(k-1)d+1}^{\min(kd,\alpha)} \mu(\|n_{\sigma(i)}\|) \right)}^{\textit{at least one sum is smaller}} \right)$$

**4.1.3 Hill-climbing Neighbourhood Generator $N_3$.** $N_3$ is designed to implement *hill climbing*. We reason as follows: If the current placement is not a global minimum, there exists a placement with at least one smaller net. Hence, to *tunnel away* from the local minimum, we generate the following neighbourhood constraints:

$$\overbrace{\bigvee_{n \in \mathbb{I}} \|n\| < \mu(\|n\|)}^{\textit{at least one net is smaller}}$$

#### 4.2 `LSSO`-based Algorithms for Placement

All the algorithms below are instantiations of Alg. 1; they use lists of neighbourhood generators, composed of the ones defined in Sect. 4.1, where hill climbing is carried out by using the neighbourhood generator $N_3$. Due to project deadline constraints, we did not explore other combinations.

1. `single_nbr_nonspec`
   (a) parameters: $\mathcal{VNS} = \bot, \mathcal{HC} = \bot, \mathcal{SPEC} = \bot, \mathcal{N}_{max} = 1$.
   (b) list of neighbourhood generators: $[N_1]$

2. `many_nbr_nonspec`
   (a) parameters: $\mathcal{VNS} = \top, \mathcal{HC} = \bot, \mathcal{SPEC} = \bot, \mathcal{N}_{max} = 10$.
   (b) list of neighbourhood generators: $N_2[\sigma, d](\mu)$, enumerated by drawing $\sigma$ and $d$ by a pseudo-random generator.
3. `many_env_spec`
   (a) parameters: $\mathcal{VNS} = \top, \mathcal{HC} = \bot, \mathcal{SPEC} = \top, \mathcal{N}_{max} = 10$.
   (b) list of neighbourhood generators: the first generator is $N_1$ and the rest are $N_2[\sigma, d](\mu)$, enumerated by drawing $\sigma$ and $d$ by a pseudo-random generator.
4. `many_env_spec_hill_clmb`
   (a) parameters: $\mathcal{VNS} = \bot, \mathcal{HC} = \top, \mathcal{SPEC} = \top, \mathcal{N}_{max} = 1$.
   (b) list of neighbourhood generators: $[N_1]$
   (c) neighbourhood generator $N_3$ is used for hill climbing.

### 5 Experimental Results

We study the performance of the following algorithms within our placement tool:

1. Algorithms which use `Polosat` as the satisfiability oracle:
   (a) `ls` (Linear Search, described in Sect. 2.3.2, with `Polosat` as the oracle)
   (b) `single_nbr_nonspec` (see Sect. 4.2)
   (c) `many_nbr_nonspec` (see Sect. 4.2)
   (d) `many_env_spec` (see Sect. 4.2)
   (e) `many_env_spec_hill_clmb` (see Sect. 4.2)
2. Algorithms which use standard SAT solving as the satisfiability oracle:
   (a) `bs_no_polosat` [19]: `OBV-BS` (see Sect. 2.3.2).
   (b) `ls_no_polosat`: Linear Search with SAT as the oracle
   (c) `many_env_spec_hill_clmb_no_polosat`: `many_env_spec_hill_clmb` with SAT instead of `Polosat` (to study the impact of disabling `Polosat` on `LSSO`, we chose `many_env_spec_hill_clmb`, since, as we shall soon see, it outperforms the other `LSSO` algorithms in a pairwise comparison).
3. `virtual-best`: represents the best result of the above algorithms per timeout.

We used an extensive set of 1200 proprietary industrial designs of various sizes and complexities. The sizes of the grids (where a *grid size* is the width $N$ multiplied by the height $M$) can be characterized as follows: a) Minimum size = 70; b) Maximum = 364000; c) Average $\approx$ 4643; d) Standard deviation $\approx$ 18829. We used machines with 32Gb of memory running Intel® Xeon® processors with 3Ghz CPU frequency.

We ran the algorithms for 600 seconds and measured the quality of the placement at different time intervals. Fig. 2 shows our main results. For each algorithm and time interval, Fig. 2 displays a score which represents the quality. The score is a real number between 0 and 1 inclusive, where the closer the score is to 1 the better. For each algorithm and time interval, the score is computed as follows: we compute the average value of the following score-per-instance: (the result of virtual-best in 600 sec.) / (the result of the current algorithm within the current time interval). Our conclusions:

First, when using SAT as the oracle, Linear Search (ls_no_polosat) outperforms OBV-BS (bs_no_polosat), demonstrating that OBV-BS is not useful when the optimization target is a complex arithmetic expression (rather than a vector of lexicographically ordered bits, where each bit is a result of a separate calculation as in [19]). Based on this result, we preferred Linear Search over OBV-BS as the baseline algorithm.

Second, confirming the conclusion of [16], Polosat makes Linear Search substantially more efficient (compare ls to ls_no_polosat).

Third, and more importantly in the context of this work, our best novel LSSO algorithm even without Polosat (many_env_spec_hill_clmb_no_polosat) is almost as efficient as Linear Search with Polosat (ls), the latter being the state-of-the-art in solving placement [16]. Moreover, the best Polosat-based LSSO algorithm (many_env_spec_hill_clmb) is significantly more efficient than both aforementioned algorithms. This result justifies the usage of both major components of our solution: LSSO–the high-level local search on top a satisfiability oracle, presented in this paper, and Polosat [16]–the low-level local search simulation with SAT.

Finally, the virtual best algorithm yields the absolutely best result, providing evidence that development of different LSSO algorithms pays off.

Additionally, Table 1 shows a pairwise comparison between our four Polosat-based LSSO algorithms. many_env_spec_hill_clmb outperforms the others.

Table 2 offers a fine-grained comparison between our best novel LSSO algorithm many_env_spec_hill_clmb and the Polosat-based Local Search ls, the latter being the state-of-the-art in solving placement [16]. The comparison is provided per grid size category and for two different timeouts. LSSO improves the performance significantly for every input size category for both timeouts. Comparing the results for the two timeouts on the biggest instances shows that increasing the timeout makes the gap between LSSO and ls more significant, given large grids.

Finally, Table 3 shows the unique contribution of each algorithm to the virtual best in 600 sec. (we dismissed all the instances on which there was more than one best-performing solver). Notably, each of the LSSO algorithms is a contributor. Surprisingly, many_nbr_nonspec contributes more than many_env_spec_hill_clmb, despite the latter algorithm outperforming the former in a pairwise comparison. A possible explanation is that we ran many_nbr_nonspec with Polosat only, while many_env_spec_hill_clmb was run twice with Polosat and SAT. Another surprising result is the significant contribution of many_env_spec_hill_clmb_no_polosat, second only to many_nbr_nonspec, implying that a SAT-based LSSO algorithm should be part of any parallel portfolio.

| | many_nbr_nonspec | single_nbr_nonspec | many_env_spec |
|---|---|---|---|
| many_env_spec_hill_clmb | (730 141 329) | (813 253 134) | (227 893 80) |
| many_nbr_nonspec | | (815 147 238) | (344 170 686) |
| single_nbr_nonspec | | | (130 280 790) |

Table 1: Pairwise comparison between LSSO algorithms for the timeout of 600 sec. Each non-empty cell $(r, c)$ contains a comparison between Algorithm $R$ in row $r$ and Algorithm $C$ in column $c$. The value (w d l) in each non-empty cell is interpreted as follows: $R$ outscored $C$ on $w$ instances; there was a draw on $d$ instances; $C$ outscored $R$ on $l$ instances.

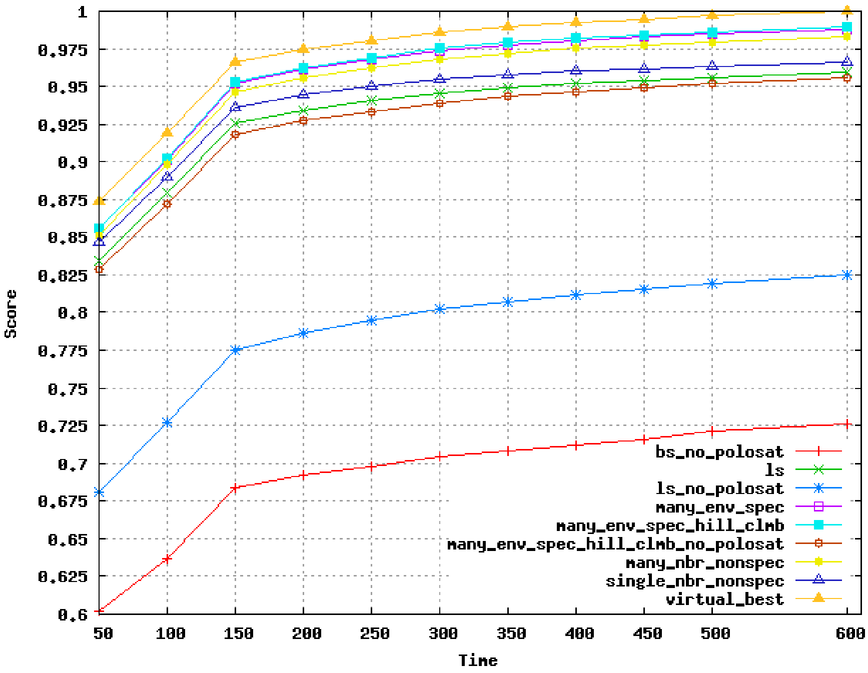

Fig. 2: Comparing Algorithms Over Time

| Grid size | Timeout of 600 seconds | | | Timeout of 300 seconds | | |
|---|---|---|---|---|---|---|
| | ls is better | Draw | LSSO is better | ls is better | Draw | LSSO is better |
| $\leq 500$ | 27 | 62 | 337 | 21 | 56 | 349 |
| $> 500$ & $\leq 10000$ | 57 | 74 | 551 | 57 | 91 | 534 |
| $> 10000$ | 17 | 28 | 47 | 18 | 40 | 34 |

Table 2: Comparing the best Polosat-based LSSO algorithm (many_env_spec_hill_clmb) to the Polosat-based Linear Search (ls), the latter comprising the previous state-of-the-art.

## 6 Conclusion

We have presented a new methodology for solving NP-hard combinatorial optimization problems, called Local Search with SAT Oracle (LSSO). Our approach can handle problems for which finding even one feasible solution is already NP-hard. LSSO applies local search which uses a SAT solver or the SAT-based optimization algorithm Polosat as an oracle. We have introduced a generic algorithm which integrates different local search schemes within the LSSO framework. Furthermore, we have implemented our approach in an industrial tool for solving the cell placement problem in VLSI and have shown that our new LSSO approach makes the tool substantially more efficient. Our tool has been successfully productized at Intel.

| Algorithm | Contribution | Algorithm | Contribution |
|---|---|---|---|
| many_nbr_nonspec | 240 | ls | 33 |
| many_env_spec_hill_clmb_no_polosat | 181 | many_env_spec | 21 |
| many_env_spec_hill_clmb | 79 | ls_no_polosat | 12 |
| single_nbr_nonspec | 54 | bs_no_polosat | 8 |

Table 3: Unique contribution to the virtual best per algorithm (sorted by the contribution).
